# A Simple Visual Estimation of Food Consumption in Carnivores

**DOI:** 10.1371/journal.pone.0034543

**Published:** 2012-05-02

**Authors:** Katherine R. Potgieter, Harriet T. Davies-Mostert

**Affiliations:** Endangered Wildlife Trust, Modderfontein, South Africa; Australian Wildlife Conservancy, Australia

## Abstract

Belly-size ratings or belly scores are frequently used in carnivore research as a method of rating whether and how much an animal has eaten. This method provides only a rough ordinal measure of fullness and does not quantify the amount of food an animal has consumed. Here we present a method for estimating the amount of meat consumed by individual African wild dogs *Lycaon pictus*. We fed 0.5 kg pieces of meat to wild dogs being temporarily held in enclosures and measured the corresponding change in belly size using lateral side photographs taken perpendicular to the animal. The ratio of belly depth to body length was positively related to the mass of meat consumed and provided a useful estimate of the consumption. Similar relationships could be calculated to determine amounts consumed by other carnivores, thus providing a useful tool in the study of feeding behaviour.

## Introduction

The use of belly-size ratings or belly scores as a method to estimate amount of food consumed by individuals has been widely utilized in predator research [1], [2], [3]. This method rates the size of an individual’s belly on a predetermined ordinal scale and gives a relative indication of whether the animal is full or hungry. A method of estimating the actual amount of meat consumed would prove productive in research on social, hunting and feeding behaviour in carnivores.

The endangered African wild dog *Lycaon pictus* is a social, cooperative, pack-living carnivore occurring in southern Africa and in parts of East Africa [4], [5], [6], [7]. Packs range between two and 50 individuals [8] and comprise a breeding pair, their dependent young and related, non-breeding helpers [9]. Wild dogs hunt communally, feed primarily on medium-sized ungulates [10] and can consume up to half their body weight in a single sitting [11]. This ability enables individuals to regurgitate portions of their meals to provide for pups or other pack members while still meeting their own nutritional needs [12].

In this study, we tested the hypothesis that the size of an individual’s belly is a reliable predictor for the amount of meat that it has consumed.

## Methods

### Study Sites and Populations

During 2006, two wild dogs were held in enclosures at the De Beers Venetia Limpopo Nature Reserve and five in enclosures at Marakele Park (Pty) Ltd in Limpopo Province, South Africa [13]. Wild dogs at both sites were habituated to being fed from a vehicle and could be individually identified by their unique coat patterns.

### Belly-size Observations

**Figure 1:**
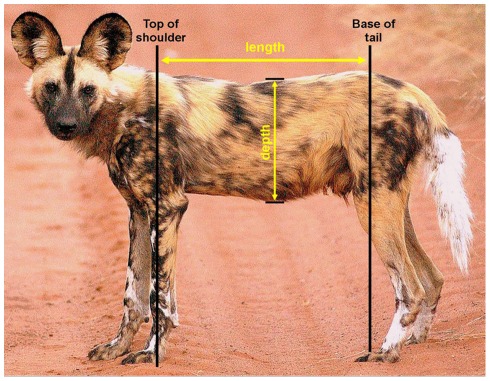
Lateral view photograph of wild dog showing how the ‘length’ and ‘depth’ measurements were taken.

We fed the seven individual wild dogs 0.5 kg pieces of meat in the enclosures. Trials began at least two full days after their previous feed to ensure that their bellies were empty. The pieces of meat were small enough to ensure that only one individual ate a single piece at a time. After each piece was eaten, a lateral photograph showing a full side view was taken with the individual standing as close to 90° to the camera as possible (Figure 1). Consecutive pieces of meat were provided until it became clear, either by lack of interest or caching of the meat, that the individual was satiated.

From each photograph we measured the *depth* from the top of the back to the lowest point on the belly and the *length* from the top of the shoulder (highest point of the scapula) to the base of the tail (highest point of the sacroiliac joint in the pelvis) (Figure 1). We then calculated the ratio of depth to length for each individual at each stage of satiation. It is important to note that, since a ratio is calculated, the measurements taken are not true dimensions but rather the individuals’ relative measurements over time. Photographs where the individual was not at, or very close to, 90° to the camera were discarded to avoid distorted measurements.

### Statistical Analysis

Multiple regression analysis (Statistica, V8, Statsoft Inc.) was used to investigate the relationship between the amount of meat consumed and belly size.

## Results and Discussion

**Figure 2:**
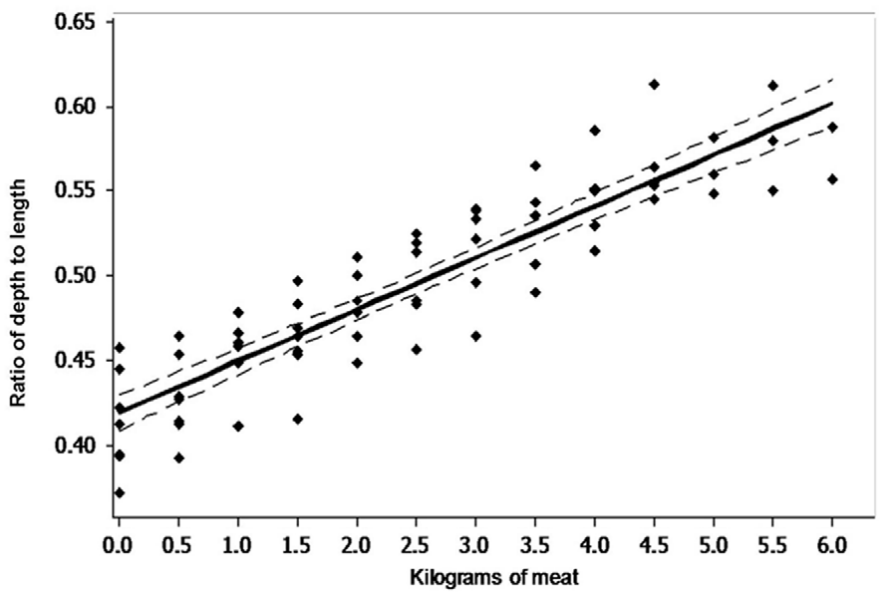
The relationship between kilograms of meat consumed and the belly depth to body length ratio (R^2^ = 0.81, n = 59, F = 299.1, p<0.001, 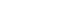). Dashed lines represent 95% confidence interval.

Individuals were fed between 0.5 and 6 kilograms, with a mean mass to satiation of 4.5 kg (n = 7). The ratio of belly depth to body length was positively related to the mass of meat consumed (R^2^ = 0.81, n = 59, F = 299.1, p<0.001, Figure 2).

The average mass of meat consumed was calculated as
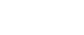
where *x* = kilograms of meat consumed, *y* = belly depth to body length ratio, the constant (0.4) represents the minimum body length:body depth value, and the coefficient (0.03) represents the slope of the relationship. Since large carnivores only occasionally eat on a fully empty stomach, the following equation was derived to account for varying degrees of initial satiation



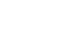
where Δx = kilograms consumed during a given time interval and Δy = change in belly ratio in the same time interval.

This correlation in wild dogs is a useful tool for the study of several aspects of wild dog feeding and provisioning behaviour. For example, knowing the approximate amount an individual has consumed can be used to: (i) assess the relative distribution of food resources among pack members; (ii) determine whether the amount consumed by an individual effects the likelihood to provision for pups; (iii) determine the effects of changes in prey density on the average amount of food consumed by individuals over time; (iv) assess the effects of seasonality on individual and/or pack consumption; and to (v) assess the effects of the density of other predators on consumption rates.

This method of determining consumption could be replicated for other species such as lion *Panthera leo,* leopard *Panthera pardus*, cheetah *Acinonyx jubatus*, and spotted hyaena *Crocuta crocuta*. A number of constraints to this method do, however, exist. Firstly, a controlled environment (i.e. captivity) is necessary to determine the baseline relationship between belly size and consumption and this may involve working with zoo or captive-bred animals that may not behave or feed in the same way as their wild, free-ranging counterparts. Secondly, the index we have presented is related to consumption on an empty stomach and corrections are likely to be necessary for species such as wild dogs that feed frequently. Wild dogs, as with many wild carnivores, rarely feed on a fully empty stomach. This is easily accounted for in our simple equation which assesses changes in belly ratio over a given time interval. Thirdly, lateral photographs must be taken at 90° to ensure accurate measurements, and this may prove difficult when operating in the field. Finally, coat length should also be short enough to enable a clear view of belly distension. Despite these potential drawbacks, this method provides a useful and simple first step in the visual quantification of consumption.
